# Supplementary material for: Structure and Dynamics of Three Escherichia coli NfsB Nitro-Reductase Mutants Selected for Enhanced Activity with the Cancer Prodrug CB1954
Source: Int J Mol Sci. 2023 Mar 22;24(6):5987. doi: 10.3390/ijms24065987 (PMC10051150; doi:10.3390/ijms24065987)
Supplement: Supplementary file 1 [file ijms-24-05987-s001.zip › ijms-2207160-supplementary/Supplementary Information-proofs/Supplementary Figure legends.pdf]

Supplementary figure legends

Supplementary Figure S1- UV/vis Spectra of wild type NfsB (left), T41L/N71S NfsB (centre) and T41Q/N71S/F124Q NfsB (right) at different redox potentials during the reduction cycle. The initial potential was -50 mV and it was decreased in 20 mV steps to -350 mV. Spectra were taken at each step. As the potential becomes more negative, the absorbance at 454 nm and 350 nm decreases.

Supplementary Figure S2- Selected distances in the minor channel to the active site in wild-type and mutant NfsB in the molecular dynamics simulations. Lanes 2-7, oxidized enzyme, Lanes 10-15 reduced enzyme. Lane 2, site 1, run 1; lane 3, site 2, run 1; lane 4, site 1, run 2, Lane 5, site 2, run 2, Lane 6, site 1 run 3, lane 7, site 2 run 3. Lane 10, site 1, run 1, lane 11 site 2, run 1; lane 12 site 1, run 2, lane 13 site 2 run 2, lane 14 site 1 run 3, lane 15 site 2 run 3. The bars show the mean distances in each run with the error bars showing the standard deviation in the distances. (a) F124-Y68 in wild type enzyme, (b) F124-Y68 in T41L/N71S, (c) F123-N67 in T41Q/N71S/F124T, (d) M127-Y68 in T41Q/N71S/F124T, (e) F123-N67 in T41Q/N71S/F124T/M127V (f) V127-Y68 in T41Q/N71S/F124T/M127V.
