# Supplementary material for: Structure and Dynamics of Three Escherichia coli NfsB Nitro-Reductase Mutants Selected for Enhanced Activity with the Cancer Prodrug CB1954
Source: Int J Mol Sci. 2023 Mar 22;24(6):5987. doi: 10.3390/ijms24065987 (PMC10051150; doi:10.3390/ijms24065987)
Supplement: Supplementary file 1 [file ijms-24-05987-s001.zip › ijms-2207160-supplementary/Supplementary Information-proofs/Supplementary Table S1- Crystal Statistics-Double and Triple mutant.pdf]

**Supplementary Table S1.**

**X-ray data collection and refinement statistics of T41L/N71S and T41Q/N71S/F124T NfsB with and without bound nicotinate.**

| **Protein** | **T41L/N71S** | **T41L/N71S with nicotinate** | **T41Q/N71S/F124T** | **T41Q/N71S/F124T with nicotinate** |
|---|---|---|---|---|
| **PDB code** | **8C5P** | **8CCV** | **8C5F** | **8C5E** |
| ***Data collection*** | | | | |
| **Wavelength (Å)** | 0.934 | 0.934 | 0.934 | 1.00 |
| **Resolution range (Å)** | 43.16-1.69 (1.74-1.69) | 47.75-2.20 (2.26- 2.20) | 47.85-1.60 (1.69-1.60) | 43.5-1.65 (1.70-1.65) |
| **Space group** | P $4_12_12$ | P $4_12_12$ | P $4_12_12$ | P $4_12_12$ |
| **Unit cell** | 57.2 Å, 57.2 Å, 263 Å 90°, 90°, 90° | 56.9 Å, 56.9 Å, 264 Å 90°, 90°, 90° | 57.2 Å, 57.2 Å, 262 Å 90°, 90°, 90° | 57.6 Å, 57.6 Å, 264 Å 90°, 90°, 90° |
| **Total reflections** | 647,523 (11,240) | 172,983 (10,839) | 345,900 (37,708) | 290,689 (18,810) |
| **Unique reflections** | 49,521 (3,011) | 15,934 (1,068) | 57,801 (8,552) | 53,617 (6,747) |
| **Multiplicity** | 13.1 (3.54) | 10.9 (10.1) | 5.9 (4.41) | 5.4, (2.79) |
| **Completeness (%)** | 99.0 (87.5) | 96.5 (77.1) | 98.6 (92.3) | 97.3 (81.4) |
| **$\langle I/\sigma I \rangle$ overall** | 36.9 (3.4) | 24.9 (5.2) | 27.2 (5.1) | 13.2 (1.9) |

| | | | | |
|---|---|---|---|---|
| **Mosaicity** | 0.096 | 0.214 | 0.112 | 0.124 |
| **Wilson B-factor (Å$^2$)** | 15.1 | 39.9 | 14.1 | 17.8 |
| **Anisotropy** | 0.339 | 0.46 | 0.071 | 0.044 |
| **Rsym[1] (%)** | 5.0 (34.8) | 7.5 (53.2) | 4.5 (29.3) | 7.8 (55.7) |
| ***Refinement and quality*** | | | | |
| **Reflections used in refinement** | 47,036 | 22,344 | 57,878 | 53,648 |
| **Reflections used for R-free** | 2,499 (5.0%) | 1,138 (5.1%) | 2,896 (5.0%) | 2,751 (5.15 %) |
| **R-work[2]** | 0.150 | 0.185 | 0.158 | 0.176 |
| **R-free[3]** | 0.188 | 0.243 | 0.181 | 0.217 |
| ***Number of non-hydrogen atoms*** | | | | |
| **macromolecules** | 3,485 | 3,352 | 3,418 | 3,402 |
| **ligands** | 114 | 80 | 114 | 84 |
| **solvent** | 545 | 167 | 500 | 517 |
| **Protein residues** | 432 | 432 | 432 | 432 |
| **RMSD bond length (Å)** | 0.015 | 0.010 | 0.014 | 0.006 |

| | | | | |
|---|---|---|---|---|
| **RMSD bond angles (°)** | 1.82 | 1.70 | 1.52 | 1.03 |
| **RMSD chirality** | 0.102 | 0.087 | 0.101 | 0.079 |
| **RMSD planarity** | 0.014 | 0.010 | 0.010 | 0.004 |
| **$F_o$-$F_c$ correlation** | 0.97 | 0.96 | 0.96 | 0.94 |
| ***Ramachandran plot*** | | | | |
| **Ramachandran favored (%)** | 99 | 98 | 99.5 | 100 |
| **Ramachandran allowed (%)** | 1 | 2 | 0.5 | 0 |
| **Ramachandran outliers (%)** | 0 | 0 | 0 | 0 |
| **Sidechain outliers (%)** | 0.8 | 2 | 0.6 | 0.8 |
| **All atom clashscore** | 3 | 1 | 2 | 1 |
| **RSRZ outliers (%)** | 0.2 | 2.5 | 0 | 2.3 |
| | | | | |
| ***Average B-factors (Å²)*** | 19.0 | 55 | 19.0 | 13.0 |
| **macromolecules** | 16 | 52 | 16 | 10 |
| **ligands** | 33.6 | 45.2 | 33.6 | 15.4 |
| **FMN** | 10 | 39.5 | 10 | 13 |

| | | | | |
|---|---|---|---|---|
| **Nicotinamide** | - | 65 | - | 20.5 |
| **solvent** | 50.7 | 49.6 | 58.1 | 55.2 |

- The numbers in parentheses represent statistics in the highest resolution shell.
- *1.* $R_{\mathrm{sym}} = \Sigma|I_{\mathrm{i}} - <|>|/\Sigma|I_{\mathrm{i}}|$ where $I_{\mathrm{i}}$ is the intensity of the $i$th measurement, and $<|>$ is the mean intensity for that reflection;
- *2.*$R_{\mathrm{work}} = \sum\|F_o| - |F_c|/\sum|F_o|$, where $F_o$ and $F_c$ are the observed and calculated structure factors for data used for refinement, respectively.
- *3.* $R_{\mathrm{free}} = \sum\|F_o| - |F_{\mathrm{c}}|/\sum|F_o|$ for 5% of the data not used at any stage of structural refinement.

**Supplementary Table S2:**

**Redox potentials of WT NfsB and mutants**.

Titrations were performed with 50-100 µM protein in 50 mM phosphate buffer, pH 7.5, 500 mM KCl, 10% glycerol, in the absence of redox mediators, with two aliquots of the same enzyme preparation. The data was fitted to either 2 single electron transfer steps (equation 1) or a concerted 2 electron transfer step (equation 2) using Sigmaplot14 with equal weighting of all points.

| **Enzyme** | **Fit to 2 single electron transfers** | | | | | | **Fit to concerted electron transfer** | | | |
|---|---|---|---|---|---|---|---|---|---|---|
| | **$E_1$ (mV)** | **P** | **$E_2$ (mV)** | **P** | **Rsqr** | **Standard error** | **Em** | **P** | **Rsqr** | **Standard error** |
| WT | -231 ± 2.8 | <0.0001 | -204 ± 2.8 | <0.0001 | 0.9975 | 0.0219 | -217.8 ± 0.7 | <0.0001 | 0.9924 | 0.0376 |
| | | | | | | | | | | |
| T41L/N71S | -215 ± 1.6 | <0.0001 | -252 ± 1.6 | <0.0001 | 0.9968 | 0.0225 | -233 ± 1.6 | <0.0001 | 0.9635 | 0.0774 |
| | | | | | | | | | | |
| T41Q/N71S/F124T | -245 ± 2.6 | <0.0001 | -215 ± 2.6 | <0.0001 | 0.9980 | 0.0194 | -230 ± 0.7 | <0.0001 | 0.9932 | 0.0354 |
| | | | | | | | | | | |

**Supplementary Table S3:**

**Stopped-flow reduction of WT and mutants by NADH and by CB1954.**

For the NADH reactions solutions containing 10 µM enzyme in one syringe and 25-1000 µM NADH in the other syringe, both in 10 mM Tris pH 7, buffer were mixed rapidly and the absorbance at 340 nm was monitored with time. The pseudo first-order rate constants were obtained by fitting the data at each concentration of substrate to an exponential decay. These were then fitted to a hyperbola in Sigmaplot 14 to obtain the kinetic parameters below. The CB1954 results are from [1].

| Enzyme | Reduction by NADH | | | | | | | | | CB1954 reduction from [1] | |
|---|---|---|---|---|---|---|---|---|---|---|---|
| | k ($s^{-1}$) | t | P | $K_d$ (µM) | t | P | $k/K_d$ ($µM^{-1} s^{-1}$) | t | P | k ($s^{-1}$) | $k/K_d$ ($µM^{-1} s^{-1}$) |
| | | | | | | | | | | | |
| WT | 1340 ± 84 | 15.8 | <0.0001 | 220 ± 30 | 7.4 | <0.0001 | 6.0 ± 0.5 | 12.6 | <0.0001 | > 30 | 0.029 ± 0.001 |
| T41L/N71S | 260 ± 11 | 25 | <0.0001 | 160 ± 15 | 11 | <0.0001 | 1.7 ± 0.1 | 17.3 | <0.0001 | > 550 | 1.6 ± 0.1 |
| T41Q/N71S/F124T | 290 ± 13 | 22 | <0.0001 | 90 ± 12 | 8 | <0.0001 | 3.0 ± 0.3 | 11.7 | <0.0001 | > 400 | 0.76 ± 0.02 |

1 Jarrom, D., Jaberipour, M., Guise, C. P., Daff, S., White, S. A., Searle, P. F. and Hyde, E. I. (2009) Steady-state and stopped-flow kinetic studies of three Escherichia coli NfsB mutants with enhanced activity for the prodrug CB1954. Biochemistry. **48**, 7665-7672

**Supplementary Table S4:**

**Selection of quadruple mutations of NfsB**

NfsB was mutated at 8 positions within the active site and the gene introduced into lambda phage. E coli lysogens were made and mutants were selected using the NfsB reduction of CB1954 to activate the SOS response and release phage enriched with more active enzyme. Amino-acid substitutions, frequency and prodrug sensitivity of mutants analysed after 15 rounds of selection:

| T41 | N71 | G120 | F124 | M127 | H128 | W138 | NUMBER | APPROX $IC_{50}$-REPLICA PLATE (μM CB1954) | $IC_{50}$ colony assay (μM CB1954) |
|---|---|---|---|---|---|---|---|---|---|
| Q | S | | T | V | | | 3 | <10 | 4.5 |
| Q | S | | T | T | | | | <10 | 5.2 |
| Q | S | | T | I | | | 2 | <10 | 5.5 |
| Q | S | | T | | | | | | 5.8 |
| Q | S | | T | Y | | | | <10 | 6.5 |
| L | S | | | F | | | 1 | <20 | 10.8 |
| L | S | | | V | | | | <20 | 10.9 |
| L | S | | | I | | | 2 | <20 | 11.3 |
| L | S | | | Y | | | | <20 | 11.3 |
| L | S | | | | | | | | 14.5 |
| L | S | S | T | L | | | 1 | <20 | nd |
| L | S | | T | T | | | 1 | <20 | nd |
| L | S | | T | Y | | | 1 | <15 | nd |
| Q | S | | | Y | | | 2 | <10 | nd |
| Q | S | S | T | L | | | | <15 | nd |

| | | | | | | | | | |
|---|---|---|---|---|---|---|---|---|---|
| Q | S | | T | S | | | | <20 | nd |
| Q | S | | | T | | | | <15 | nd |
| | S | | T | | | | | 20 | nd |

**Supplementary Table S5:**

**Steady-state kinetics parameters for the reduction of CB1954 by NADH catalysed by NfsB mutants.**

Initial rates were monitored at 420 nm at a series of concentrations of both substrates and all the data fitted to the equation for a bi-bi substituted enzyme reaction (equation4), by nonlinear regression with equal weighting all points, using the programme Sigmaplot 14 (Systat software, San Jose, CA). The data for N71S/T41Q/F124T/M127V is from this work. The data for the other mutants is from [1]

| **Protein** | $k_{cat}$ $s^{-1}$ | $K_{mCB1954}$ µM | $k_{cat}/K_{mCB1954}$ $\mu M^{-1} s^{-1}$ | $K_{mNADH}$ µM | $k_{cat}/K_{mNADH}$ $\mu M^{-1} s^{-1}$ |
|---|---|---|---|---|---|
| N71S/T41Q/F124T/M127V | 270 ± 17 | 480 ± 60 | 0.57 ± 0.04 | 100 ± 13 | 2.7 ± 0.2 |
| Wild type | 140 ± 32 | 17200 ± 4800 | 0.007 ± 0.0006 | 40 ± 12 | 3.46 ± 0.6 |
| T41L/N71S | 153 ± 8 | 216 ± 33 | 0.71 ± 0.08 | 253 ± 41 | 0.62 ± 0.08 |
| T41Q/N71S/F124T | 181 ± 7 | 569 ± 45 | 0.32 ± 0.02 | 136 ± 12 | 1.33 ± 0.077 |


1 Jarrom, D., Jaberipour, M., Guise, C. P., Daff, S., White, S. A., Searle, P. F. and Hyde, E. I. (2009) Steady-state and stopped-flow kinetic studies of three Escherichia coli NfsB mutants with enhanced activity for the prodrug CB1954. Biochemistry. **48**, 7665-7672

**Supplementary Table S6.**

**X-ray data collection and refinement statistics of T41Q/N71S/F124T/M127V NfsB bound to nicotinate.**

| **PDB code** | **8CJ0** |
|---|---|
| ***Data collection*** | |
| **Wavelength (Å)** | 0.934 |
| **Resolution range (Å)** | 35.06-1.99 (2.05-1.99) |
| **Space group** | P $4_1 2_1 2$ |
| **Unit cell** | 57.3 Å, 57.3 Å, 266 Å 90°, 90°, 90° |
| **Total reflections** | 290,689 (19,436) |
| **Unique reflections** | 31,129 (2,176) |
| **Multiplicity** | 9.0 (8.93) |
| **Completeness (%)** | 98.9 (95.3) |
| **$\langle I/\sigma I \rangle$ overall** | 25.3 (6.2) |
| **Mosaicity** | 0.141 |
| **Wilson B-factor (Å$^2$)** | 25.9 |

| | |
|---|---|
| **Anisotropy** | 0.167 |
| **Rsym[1] (%)** | 7.5 (55.0) |
| ***Refinement and quality*** | |
| **Reflections used in refinement** | 31,128 (2168) |
| **Reflections used for R-free** | 1,575 (5.1%) |
| **R-work[2]** | 0.170 |
| **R-free[3]** | 0.205 |
| ***Number of non-hydrogen atoms*** | 3622 |
| **macromolecules** | 3344 |
| **ligands** | 84 |
| **solvent** | 155 |
| **Protein residues** | |
| **RMSD bond length (Å)** | 0.011 |
| **RMSD bond angles (°)** | 1.28 |
| **RMSD chirality** | 0.067 |
| **RMSD planarity** | 0.007 |

| | |
|---|---|
| **$F_o$-$F_c$ correlation** | 0.96 |
| ***Ramachandran plot*** | |
| **Ramachandran favored (%)** | 99.5 |
| **Ramachandran allowed (%)** | 0.5 |
| **Ramachandran outliers (%)** | 0 |
| **Sidechain outliers (%)** | 1.7 |
| **All atom clash score** | 2 |
| **RSRZ outliers (%)** | 4.6 |
| | |
| ***Average B-factors ($Å^2$)*** | 34.0 |
| **macromolecules** | 36 |
| **ligands** | |
| **FMN** | 20 |
| **Nicotinamide** | 42 |
| **solvent** | 46.4 |

- The numbers in parentheses represent statistics in the highest resolution shell.

- *1.* $R_{\mathrm{sym}} = \Sigma|I_{\mathrm{i}} - <|>|/\Sigma|I_{\mathrm{i}}|$ where $I_{\mathrm{i}}$ is the intensity of the *i*th measurement, and $<|>$is the mean intensity for that reflection;
- *2.*$R_{\mathrm{work}} = \sum\|F_o| - |F_c|/\sum|F_o|$, where $F_o$ and $F_c$ are the observed and calculated structure factors for data used for refinement, respectively.
- *3.* $R_{\mathrm{free}} = \sum\|F_o| - |F_{\mathrm{c}}|/\sum|F_o|$ for 5% of the data not used at any stage of structural refinement.
