# Supplementary material for: Structure and Dynamics of Three Escherichia coli NfsB Nitro-Reductase Mutants Selected for Enhanced Activity with the Cancer Prodrug CB1954
Source: Int J Mol Sci. 2023 Mar 22;24(6):5987. doi: 10.3390/ijms24065987 (PMC10051150; doi:10.3390/ijms24065987)
Supplement: Supplementary file 1 [file ijms-24-05987-s001.zip › ijms-2207160-supplementary/Supplementary Information-proofs/Supplementary Table S2- redox data.pdf]

Supplementary Table S2:

Redox potentials of WT NfsB and mutants.

Titrations were performed with 50-100 μM protein in 50 mM phosphate buffer, pH 7.5, 500 mM KCl, 10% glycerol, in the absence of redox mediators, with two aliquots of the same enzyme preparation. The data was fitted to either 2 single electron transfer steps (equation 1) or a concerted 2 electron transfer step (equation 2) using Sigmaplot14 with equal weighting of all points.

Enzyme	Fit to 2 single electron transfers						Fit to concerted electron transfer			
	E_1 (mV)	P	E_2 (mV)	P	Rsqr	Standard error	Em	P	Rsqr	Standard error
**WT**	-231 ± 2.8	<0.0001	-204 ± 2.8	<0.0001	0.9975	0.0219	-217.8 ± 0.7	<0.0001	0.9924	0.0376
**T41L/N71S**	-215 ± 1.6	<0.0001	-252 ± 1.6	<0.0001	0.9968	0.0225	-233 ± 1.6	<0.0001	0.9635	0.0774
**T41Q/N71S/F124T**	-245 ± 2.6	<0.0001	-215 ± 2.6	<0.0001	0.9980	0.0194	-230 ± 0.7	<0.0001	0.9932	0.0354
