# Supplementary material for: Structure and Dynamics of Three Escherichia coli NfsB Nitro-Reductase Mutants Selected for Enhanced Activity with the Cancer Prodrug CB1954
Source: Int J Mol Sci. 2023 Mar 22;24(6):5987. doi: 10.3390/ijms24065987 (PMC10051150; doi:10.3390/ijms24065987)
Supplement: Supplementary file 1 [file ijms-24-05987-s001.zip › ijms-2207160-supplementary/Supplementary Information-proofs/Supplementary Table S3- stopped flow data.pdf]

Supplementary Table S3:

Stopped-flow reduction of WT and mutants by NADH and by CB1954.

For the NADH reactions solutions containing 10 µM enzyme in one syringe and 25-1000 µM NADH in the other syringe, both in 10 mM Tris pH 7, buffer were mixed rapidly and the absorbance at 340 nm was monitored with time. The pseudo first-order rate constants were obtained by fitting the data at each concentration of substrate to an exponential decay. These were then fitted to a hyperbola in Sigmaplot 14 to obtain the kinetic parameters below. The CB1954 results are from [1].

Enzyme	Reduction by NADH									CB1954 reduction from [1]	
	k (s^{-1})	t	P	K_d (µM)	t	P	k/K_d ($µM^{-1} s^{-1}$)	t	P	k (s^{-1})	k/K_d ($µM^{-1} s^{-1}$)
WT	1340 ± 84	15.8	<0.0001	220 ± 30	7.4	<0.0001	6.0 ± 0.5	12.6	<0.0001	> 30	0.029 ± 0.001
T41L/N71S	260 ± 11	25	<0.0001	160 ± 15	11	<0.0001	1.7 ± 0.1	17.3	<0.0001	> 550	1.6 ± 0.1
T41Q/N71S/F124T	290 ± 13	22	<0.0001	90 ± 12	8	<0.0001	3.0 ± 0.3	11.7	<0.0001	> 400	0.76 ± 0.02

1 Jarrom, D., Jaberipour, M., Guise, C. P., Daff, S., White, S. A., Searle, P. F. and Hyde, E. I. (2009) Steady-state and stopped-flow kinetic studies of three Escherichia coli NfsB mutants with enhanced activity for the prodrug CB1954. Biochemistry. **48**, 7665-7672
