# Supplementary material for: Structure and Dynamics of Three Escherichia coli NfsB Nitro-Reductase Mutants Selected for Enhanced Activity with the Cancer Prodrug CB1954
Source: Int J Mol Sci. 2023 Mar 22;24(6):5987. doi: 10.3390/ijms24065987 (PMC10051150; doi:10.3390/ijms24065987)
Supplement: Supplementary file 1 [file ijms-24-05987-s001.zip › ijms-2207160-supplementary/Supplementary Information-proofs/Supplementary Table S4-selection of quadruple mutants.pdf]

Supplementary Table S4:

Selection of quadruple mutations of NfsB

NfsB was mutated at 8 positions within the active site and the gene introduced into lambda phage. E coli lysogens were made and mutants were selected using the NfsB reduction of CB1954 to activate the SOS response and release phage enriched with more active enzyme.

Amino-acid substitutions, frequency and prodrug sensitivity of mutants analysed after 15 rounds of selection:

T41	N71	G120	F124	M127	H128	W138	NUMBER	APPROX IC_{50}- REPLICA PLATE (µM CB1954)	IC_{50} colony assay (µM CB1954)
Q	S		T	V			3	<10	4.5
Q	S		T	T				<10	5.2
Q	S		T	I			2	<10	5.5
Q	S		T						5.8
Q	S		T	Y				<10	6.5
L	S			F			1	<20	10.8
L	S			V				<20	10.9
L	S			I			2	<20	11.3
L	S			Y				<20	11.3
L	S								14.5
L	S	S	T	L			1	<20	nd
L	S		T	T			1	<20	nd
L	S		T	Y			1	<15	nd
Q	S			Y			2	<10	nd
Q	S	S	T	L				<15	nd
Q	S		T	S				<20	nd
Q	S			T				<15	nd
	S		T					20	nd
