# Supplementary material for: Structure and Dynamics of Three Escherichia coli NfsB Nitro-Reductase Mutants Selected for Enhanced Activity with the Cancer Prodrug CB1954
Source: Int J Mol Sci. 2023 Mar 22;24(6):5987. doi: 10.3390/ijms24065987 (PMC10051150; doi:10.3390/ijms24065987)
Supplement: Supplementary file 1 [file ijms-24-05987-s001.zip › ijms-2207160-supplementary/Supplementary Information-proofs/Supplementary Table S5- steady state kinetics of mutants.pdf]

Supplementary Table S5

Steady-state kinetics parameters for the reduction of CB1954 by NADH catalysed by NfsB mutants.

Initial rates were monitored at 420 nm at a series of concentrations of both substrates and all the data fitted to the equation for a bi-bi substituted enzyme reaction (equation4), by nonlinear regression with equal weighting all points, using the programme Sigmaplot 14 (Systat software, San Jose, CA). The data for N71S/T41Q/F124T/M127V is from this work. The data for the other mutants is from [1]

**Protein**	k_{cat} s^{-1}	$K_{mCB1954}$ μM	$k_{cat}/K_{mCB1954}$ $\mu M^{-1} s^{-1}$	K_{mNADH} μM	k_{cat}/K_{mNADH} $\mu M^{-1} s^{-1}$
N71S/T41Q/F124T/M127V	270 ± 17	480 ± 60	0.57 ± 0.04	100 ± 13	2.7 ± 0.2
Wild type	140 ± 32	17200 ± 4800	0.007 ± 0.0006	40 ± 12	3.46 ± 0.6
T41L/N71S	153 ± 8	216 ± 33	0.71 ± 0.08	253 ± 41	0.62 ± 0.08
T41Q/N71S/F124T	181 ± 7	569 ± 45	0.32 ± 0.02	136 ± 12	1.33 ± 0.077

1 Jarrom, D., Jaberipour, M., Guise, C. P., Daff, S., White, S. A., Searle, P. F. and Hyde, E. I. (2009) Steady-state and stopped-flow kinetic studies of three Escherichia coli NfsB mutants with enhanced activity for the prodrug CB1954. Biochemistry. **48**, 7665-7672
