# Supplementary material for: Structure and Dynamics of Three Escherichia coli NfsB Nitro-Reductase Mutants Selected for Enhanced Activity with the Cancer Prodrug CB1954
Source: Int J Mol Sci. 2023 Mar 22;24(6):5987. doi: 10.3390/ijms24065987 (PMC10051150; doi:10.3390/ijms24065987)
Supplement: Supplementary file 1 [file ijms-24-05987-s001.zip › ijms-2207160-supplementary/Supplementary Information-proofs/Supplementary Table S6- Crystal Statistics-Quadruple mutant.pdf]

Supplementary Table S6.

X-ray data collection and refinement statistics of T41Q/N71S/F124T/M127V NfsB bound to nicotinate.

**Protein**	**T41Q/N71S/F124T/M127V NfsB**
**PDB code**	**8CJ0**
Data collection	
**Wavelength (Å)**	0.934
**Resolution range (Å)**	35.06-1.99 (2.05-1.99)
**Space group**	P $4_1 2_1 2$
**Unit cell**	57.3 Å, 57.3 Å, 266 Å 90°, 90°, 90°
**Total reflections**	290,689 (19,436)
**Unique reflections**	31,129 (2,176)
**Multiplicity**	9.0 (8.93)
**Completeness (%)**	98.9 (95.3)
**$\langle I/\sigma I \rangle$ overall**	25.3 (6.2)

**Mosaicity**	0.141
**Wilson B-factor (Å^2)**	25.9
**Anisotropy**	0.167
**Rsym[1] (%)**	7.5 (55.0)
Refinement and quality	
**Reflections used in refinement**	31,128 (2168)
**Reflections used for R-free**	1,575 (5.1%)
**R-work[2]**	0.170
**R-free[3]**	0.205
Number of non-hydrogen atoms	3622
**macromolecules**	3344
**ligands**	84
**solvent**	155
**Protein residues**	
**RMSD bond length (Å)**	0.011
**RMSD bond angles (°)**	1.28

**RMSD chirality**	0.067
**RMSD planarity**	0.007
**F_o-F_c correlation**	0.96
Ramachandran plot	
**Ramachandran favored (%)**	99.5
**Ramachandran allowed (%)**	0.5
**Ramachandran outliers (%)**	0
**Sidechain outliers (%)**	1.7
**All atom clash score**	2
**RSRZ outliers (%)**	4.6
Average B-factors ($Å^2$)	34.0
**macromolecules**	36
**ligands**	
**FMN**	20
**Nicotinamide**	42

**solvent**	46.4

- The numbers in parentheses represent statistics in the highest resolution shell.
- *1.* $R_{sym} = \Sigma|I_i - <|>|/\Sigma|I_i|$ where I_i is the intensity of the *i*th measurement, and <|>is the mean intensity for that reflection;
- *2.*$R_{work} = \sum\|F_o| - |F_c|/\sum|F_o|$, where F_o and F_c are the observed and calculated structure factors for data used for refinement, respectively.
- *3.* $R_{free} = \sum\|F_o| - |F_c|/\sum|F_o|$ for 5% of the data not used at any stage of structural refinement.
